# A Hypothesis of Gender Differences in Self-Reporting Symptom of Depression: Implications to Solve Under-Diagnosis and Under-Treatment of Depression in Males

**DOI:** 10.3389/fpsyt.2021.589687

**Published:** 2021-10-25

**Authors:** Peixia Shi, Aigang Yang, Qing Zhao, Zhaohua Chen, Xiaomei Ren, Qin Dai

**Affiliations:** ^1^Department of Nursing Psychology, Army Medical University, Chongqing, China; ^2^CAS Key Laboratory of Mental Health, Institute of Psychology, Chinese Academy of Sciences (CAS), Beijing, China; ^3^Department of Psychology, University of Chinese Academy of Sciences, Beijing, China

**Keywords:** gender difference, male depression, suicide, self-reporting symptom, coping style, symptom phenotype

## Abstract

The phenomenon of female preponderance in depression has been well-reported, which has been challenged by higher rates of suicide and addictive behaviors in males, and a longer life-span in females. We thus propose an alternative hypothesis “Gender differences in self-reporting symptom of depression,” suggesting mild-moderate depression tends to be reported more often by females, and severe depression and suicide tend to be reported more often by males. Potential mechanisms that account for this difference may include three aspects: covariation between estrogen levels and the incidence peak of female depression, gender differences in coping style (e.g., comparative emotional inexpressiveness and non-help-seeking in males), and gender differences in symptom phenotypes (e.g., atypical symptoms in male depression). Our newly presented hypothesis implied the overlooked under-diagnosis and under-treatment of depression in males. For effective diagnoses and timely treatment of male depression, it is critical to incorporate symptoms of depression in males into the relevant diagnostic criteria, encourage males to express negative emotions, and increase awareness of suicidal behavior in males.

## Introduction

In the past several decades, gender differences in depression have been extensively discussed. A few studies have found the gender difference in depression to be small or absent (1), and no gender difference has been indicated in psychotic or melancholic depression (2). However, most studies have confirmed that depression is twice as common in women than in men (3, 4), which has been reported across different cultures (5). Depression is disproportionately reported by women (almost twice as often as by men) during reproductive age (3, 6–8). For example, the worldwide annual prevalence of depression in 2010 for females and males was 5.5 and 3.2%, respectively (i.e., 1.72 vs. 1) (9, 10). In Canada, the prevalence was 5.0% in women and 2.9% in men in 2002 (i.e., 1.72 vs. 1), and it increased to 5.8% in women and 3.6% in men in 2012 (i.e., 1.61 vs. 1) (11). In the USA, women had an ~2-fold higher risk of depression than men, with 21.3% of women and 12.9% of men experiencing major depressive episodes during their lifetimes (12). In a cross-sectional study of Pakistan, the majority (78.9%) of people diagnosed with major depression were women (13). Consistently, a review of studies between 1994 and 2014 with community participants from 30 countries showed that the point prevalence of depression in the community was significantly higher in females (14.4%) compared with males (11.5%) (i.e., 1.25 vs. 1) (14).

Even in specific populations, a female preponderance of depression has been confirmed. Among students of pedagogy, 9.42% of females reported depression, compared with 1.23% males (i.e., 7.66 vs. 1) (15). In Polish adolescents, being female was reportedly a major risk factor for depression (16). In a study of individuals with diabetes, the prevalence of comorbid depression was significantly higher in women (28%) than in men (18%) (i.e., 1.56 vs. 1) (17), which was further confirmed by a later review (18). In a similar cross-sectional study conducted in a gastroenterology clinic, compared with males, females reported more symptoms of depression (44 vs. 32%) (i.e., 1.38 vs. 1) (19). In brief, women have reported depression and been diagnosed with depression substantially more often than men (5, 20, 21).

## Challenges for the Female Preponderance Hypothesis

Although women evidently tend to report depression more than men (3, 4), several observations raise questions as to the root cause of this, e.g., the higher suicide rate in males (22), longer life-span in females (23), and greater rates of alcoholism and other addictive behaviors in males (24).

With regard to suicide rates which correlates with age, gender, and socioeconomic status (25), although females reported significantly higher rates of suicidal ideation (OR 1.32) (26); however, the ratio of male to female completed suicides was 1.97:1 (27). It has also been reported that compared with women, men were three times more likely to die from suicide (28). Moreover, in one study, compared with patients who attempted suicide unsuccessfully, those who successfully committed suicide were more likely to be male (29). The finding inspired the argument that higher suicide rates in males may result from the more lethal methods used by males to attempt suicide (30). However, in this study (29), attempted suicide was defined as “acts of self-inflicted injury or self-poisoning with overdose drugs,” which should be more accuracy with the term of “self-harm behavior.” Notably, in a recent review, the ratio of male to female non-fatal attempted suicides was 1.21:1 (27). Indeed, being male is reported significantly associated with all types of suicidal behavior (31). The gender ratio in suicide behavior persisted even in the elder sample (2.74:1) aged above 60 (32). Importantly, higher suicide rates are associated with more severe depression (33, 34). Without regard for age and socioeconomic status, if the female preponderance hypothesis is true, these aforementioned observations suggest that women suffer from depression more but suicide less, whereas men suffer from depression less but suicide more. This can-not be simply explained by weaker suicide intention in female depression due to their social responsibility, and is conflicted with the fact that over 90% of people who die by suicide had a psychiatric disease, mainly depression (35). It may be that males with mild depression are less likely to express it and seek help, which results in more suicide causing by depression in males (36).

Another challenge for the female preponderance hypothesis is the longer life-span of females. Historically, women live longer than men in almost every country in the world (23). The life expectancy at birth is 79.3 years for females (86.3 in Japan) and 71.9 years for males (78.3 in Japan) (37). A recent review concluded that in developed countries women live ~4–7 years longer than men (38). Despite the complex interaction of environmental, historical, and genetic factors on age-related diseases and longevity between genders (37), however, if the female preponderance hypothesis is true, these results suggest that females suffer from depression more and live longer. This challenges our understanding of depression because that depression is detrimental to physical health (39, 40), and is associated with a higher morbidity rate (41). It has also recently been reported that even in depressed subpopulations, mortality was higher in men than in women (42). Obviously, high morbidity in males can-not attribute to pure genetic factors or life-style, since that particular life-style (e.g., addiction and aggression) is highly correlated with depression in males (43, 44). Thus, the truth might be that females tend to express more depressive symptoms (45, 46) as a way of help-seeking, even they perceive similar symptoms as male (47).

A third challenge for the female preponderance hypothesis is the greater rate of addictive behaviors in males such as alcoholism and substance use disorder. Some studies indicated that gender difference is not observed as to the intensity of internet dependence (48). However, a gender difference in the risk for developing an addictive behavior was confirmed, with a significantly higher risk in males for several addiction tendencies (49), including alcohol dependence/abuse (48.1 vs. 24.5%) (24), substance use disorder (50) and tobacco dependence (51). Notably, the presence of either depression or alcoholism doubled the risks for another (OR range from 2.00 to 2.09) (52). Nearly one-third of patients with the major depressive disorder also suffered from substance use disorders, which yielded a higher risk of suicide (53). Thus, if the female preponderance hypothesis is true, these results suggest that males suffer from depression less, but report alcoholism, substance use disorder or other addictive behaviors more. This prompts the question as to what causes men to be more prone to addictive behavior. In a previous study, the incidence of depression was equal in males and females after controlling for alcoholism (54). Therefore, addictive behaviors in males may result in under-diagnosis of male depression, because that addictive behavior is not a typical symptom of depression and it can mask traditional symptoms of depression (55).

The three phenomena listed above strongly challenge the female preponderance hypothesis of depression, and suggest a need for a more comprehensive hypothesis. Herein we proposed an alternative hypothesis—gender differences in self-reporting symptom of depression, in an effort to better reconcile the numerous different observations and forms of evidence together.

## Gender Differences in Self-Reporting Symptom of Depression Hypothesis

**Figure 1:**
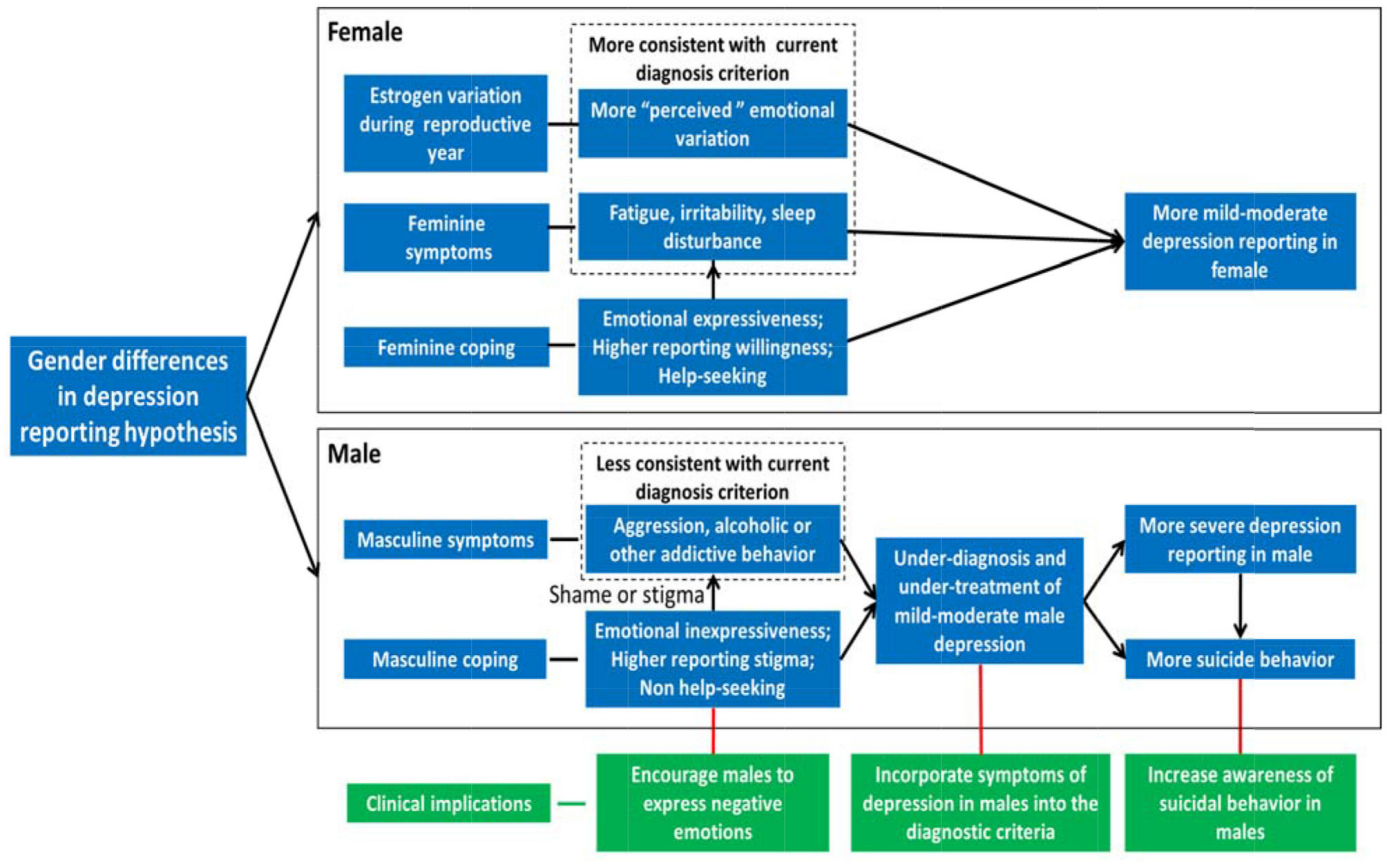
Gender differences in self-reporting symptom of depression hypothesis. For females, estrogen variation during the reproductive year in female leads to more “perceived” emotional variation in the woman. Meanwhile, feminine coping styles (e.g., help-seeking and emotional expressiveness) and social expectations for females lead to traditional female depressive symptoms (e.g., fatigue, irritability, and sleep disturbance), which are more consistent with the current diagnosis criterion of depression. As a result, more mild-moderate depression is diagnosed in females. For males, masculine coping style (e.g., non-help-seeking and emotional inexpressiveness) and social expectation for males lead to more shame or stigma to express depression, results in traditional male depressive symptoms (e.g., aggression, alcohol, or substance abuse), which are less consistent with current diagnosis criterion of depression. As a result, mild-moderate male depression is under-diagnosed and under-treated, which leads to more severe depression and higher suicide reporting in males, and needs immediate attention. To diagnose and treat male depression early and effectively, it is critical to incorporate symptoms of depression in males into the relevant diagnostic criteria, encourage males to express negative emotions, and increase awareness of suicidal behavior in males.

The basic tenets of the gender differences in depression severity reporting hypothesis (Figure 1) are that women are more likely to report mild-moderate symptoms of depression (56), and men intend to report more severe depression (56, 57) and higher suicide (28, 31).

### More Mild-Moderate Symptom Reporting in Females

Females evidently exhibited a greater tendency to recognize subtle emotional changes than males, alternatively, they may have actually “perceived” more emotional symptoms (45). Similarly, female patients with depression reported more emotional experiences, particularly negative emotional experiences, than male patients (58). Therefore, females constantly reported more mild-moderate depression across all age bands (59). As a result, more mild-moderate depression was reported and diagnosed in females, whereas mild-moderate male depression was under-reported and under-diagnosed (56). Consistently, a gender difference was only significant when including minor depression based on of the general Danish population (60), suggesting that the female preponderance is more pronounced in less severe depression status. Study also suggested that amongst patients with severe depression, the gender ratio of patients was no longer significant (i.e., the female preponderance in depression was decreased along with the severity of depression) (59). Thus, in a recent report, it was recommended that optimal cut-off points for depression should be much higher for females (19/20, sensitivity 74.5% and specificity 73.8%) than for males (13/14, sensitivity 72.2% and specificity 64.1%) (61). Hence, with equal depression cut-off scores for both males and females, more women would report symptoms and reach the diagnostic criteria for depression, and male depression would be less likely to be detected and diagnosed.

### More Severe Depression and Higher Suicide Reporting in Males

When untreated, 53% mild to moderate depression will remit within 12 months (62). Meanwhile, untreated mild-moderate depression leads to a high immediate and subsequent suicide risk (63), which is also the largest risk factor for suicide (64). Compared with women, men are less likely to sought treatment due to the shame of seeking help for hegemonic masculinity (65). Under-diagnosis and under-treatment of male depression lead to prolonged depression and higher a suicide rate in men (65, 66). In one recent study, experiencing social pressure not to express negative feelings predicted increases in symptoms of depression, which may be particularly true in males due to their hegemonic masculinity (67). In another study, when required to be dependent on others, men exhibited more severe depression than women (57), in which 58% of depression occurred in men. Indeed, severe depression was not significantly differed between genders (59), indicating a higher male/female reporting ratio of severe depression compared with the gender ratio of reporting mild-moderate depression.

Higher suicide rates are associated with more severe depression (33, 34). Despite the report of gender paradox in suicidal behavior (26, 68, 69): Over representation of females in non-fatal suicidal behavior and a preponderance of males in committed suicide. However, females are only over-represented in suicidal ideation (26, 69) or self-harm behavior (70). In fact, the ratios of male to female attempted suicides and completed suicides were 1.21:1 and 1.97:1, respectively (27). The gender ratio of attempted suicide was even higher in adolescent (2.07:1) (71), while the suicidal commitment risk continued to elders aged 60 (2.74:1) (32). Depressed men often experience a loss of control, a hidden self, and substance use or abuse, which may cause them to commit suicide as a definitive means of eliminating their sense of a loss of control (72). In a previous study, suicide rates were significantly negatively correlated with rates of treatment for depression (73), i.e., higher suicide rates were associated with lower treatment rates. The above-described results suggest that men report more severe depression and higher suicide rates than women (56, 65, 66, 74), which may result from lower rates of diagnosis and treatment of male depression (36).

## Potential Mechanisms

### Biological Dimension: Covariation Between Estrogen Levels and Incidence Peak of Female Depression

Rates of depression in males and females vary during their life-spans. Before puberty, girls and boys have similar rates of depression or a slight higher in boys (3). The prevalence of depression tends to be doubled in girls (3, 6, 75, 76) from the age of 12 years, and this trend persists until the age of 45 or 54 years (26, 77), then declines after menopause (3, 8, 78). Although one study indicated that the gender difference in depression persisted in elders (79), most studies reported that at ages > 65 years, both men and women exhibit declines in depression rate, which becomes similar between them again (11, 76, 80, 81). These results indicate that the “female preponderance in depression” could be relevant to the sex hormonal level (e.g., estrogen).

Longitudinal studies indicate that as soon as estrogen levels rise (first menstruation of girls) (82), the rate of major depression in girls increases in tandemly (83). Consistently, the peak incidence of depression during childbearing years is reportedly associated with cyclic estrogen changes, with a higher prevalence of depression in females at the premenstrual stage, during pregnancy, and at postpartum and peri-menopausal stages (84). About 48% of females who suffer from premenstrual syndrome (85) reported a depressed mood and fatigue in the week before the onset of menstruation (86). Consistently, during the menopausal transition, when sex hormones strongly fluctuated, “depressed mood” and “sleeping problems” were common complaints (87). In summary, the incidence of female depression evidently fluctuates with estrogen levels, leading to more “perceived” fluctuation in mood and more reporting of depression in female.

### Psychological Dimension: Gender Differences in Coping Styles

Another explanation for the female preponderance of depression is that women show a more feminine coping style, and they are more willing to express affective symptoms and seek medical help (88). As previously introduced, women evidently report more mild depression whereas men report more severe depression (56). In one study, females reported more symptoms of depression than males (44 vs. 32%), and were more likely to subsequently seek help at private clinics (23 vs. 14%) or from a Quran therapist (11 vs. 5%) (19). Even in a 70-year-old population, femininity was associated with higher levels of depression (89).

In contrast, the masculine coping style was being emotionally unexpressive (90) and reluctant to seek help (91). Indeed, males reported less depression, even they experienced more intense emotions (92), indicating that they need greater symptom severity to ask for help. The results also suggested that males intend to “omit” symptoms, while females “notice” symptoms. Consequently, females may start to report depressed moods with mild or moderate severity, while males might only begin to report depression with much severer severity (56). Similarly, it has been reported that men were more likely to forget episodes that had generally not reached “case” criteria, whereas women were more likely to remember them (46). Due to the shame of seeking help or showing weakness, males tend to hide symptoms of depression from people and try hard to appear cheerful and exhibit happiness in the presence of others (93). Even after stratification by clinically significant impairment and paid employment status, men reported fewer symptoms of depression than women, and as a result men reached the diagnostic threshold less often (47). Instead, they tended to mask symptoms of depression, leading providers to under-diagnose and under-treat men for depression (94–96). Even though males seek help, they intend to report fewer symptoms and low severity to maintain masculine status (97, 98). Thus, gender differences in coping style may have resulted in a “masculine” form of depression in the general population that is under-diagnosed and under-treated (99).

### Social Dimension: Gender Differences in Phenotypic Symptoms of Depression

Hegemonic masculinity indicates how a gender role is enacted with depression expression, in which social expectation for males was proactive, aggression, and violent, while female stereotype was affective, passive, and selfless (90). Thus, although no evidence for a gender-related somatic factor was reported in one study (100), and the lower rate of poor appetite (OR 0.69) in females was indicated in another study (26). Most studies indicated a female predominance not in “pure depression” but in a specific phenotype in women, i.e., “somatic depression” (appetite, sleep, and fatigue) (43, 44, 101), which is generally consistent with current diagnostic criteria for depression. The female rates were consistently higher across all age bands only in DSM-IV mood disorder, major depressive disorder and non-melancholic mood disorder (59). In contrast, men exhibit more atypical signs of depression such as aggression and antisocial behaviors (102). In a study of 18,807 Korean, female depression was significantly associated with fatigue, hypersomnia, and psychomotor retardation (103). In another study, women commonly reported concurrent symptoms consistent with anxiety disorders, somatoform disorder, and bulimia, whereas drug and alcohol abuse was more common in men (104). Relatively, comorbid anxiety was reportedly more prevalent in women, whereas comorbid alcohol abuse was a major concern in men (20). In conclusion, women evidently exhibit more symptoms of fatigue, irritability, and sleep disturbance (somatic depression) which are consistent with current diagnostic criteria for depression, whereas men exhibit more atypical symptoms of aggression or substance addiction which are less consistent with current diagnostic criteria for depression (44, 105, 106). Thus, it is reasonable to assume that this results in comparatively less diagnosis and treatment of male depression.

The social role of male, especially hegemonic masculinity guaranteed that male depression might manifest as substance abuse, aggressive, and/or violent practices (107). Due to the fact that for males showing weakness is contrary to social expectations (being strong, independent, and exhibiting self-control), depressive symptoms (e.g., tiredness and weakness) bring men more shame or stigma to admit or seek help (108). Additional evidence is concordant with a “hegemonic” view—particularly concerning independence—that men “should” be reluctant to seek help; in fact they tend to repress symptoms of depression and/or hide them from others (109). Research suggests that men who are depressed may experience a trajectory of emotional distress that results in avoidant, numbing, and escapist behavior that can lead to substance use, aggression, violence, and suicide (110). Constantly, males exhibit significantly higher rates of substance use and physical violence (111, 112). Gender roles, in particular hegemonic masculinity, may primarily influence the expression of depression rather than the actual experience of depression *per se*, and this may in turn contribute to under-diagnosis and under-treatment of male depression.

## Clinical Implications of the Gender Differences in Self-Reporting Symptom of Depression Hypothesis

The principal value of the above newly proposed hypothesis is to emphasize that male depression is under-diagnosed and under-treated, which requires immediate attention and action. The critical implication of this theory is summarized in the following three points.

### Incorporating Symptoms of Depression in Males Into Current Diagnostic Criteria

Differential symptoms of depression in males and females (28) and low diagnosis and treatment rates of male depression (29, 54) suggest a greater need to improve the current screening for depression. Evidence suggests that standard assessments of depression omit several key components of male depression, mainly substance use and violence (107). Instead, a combination of the Patient Health Questionnaire and the Gotland Male Depression Scale (to be introduced^1^) may facilitate a more sensitive and accurate identification of male depression (116). It has been suggested that using gender-sensitive assessment strategies would assure that more men would be identified and treated for depression (117). In a study that utilized these alternative male-oriented diagnostic tools, the prevalence of depression was higher in men than in women (112). Notably, the results of another study suggest that male-type depressive symptoms may also be highly prevalent in females (118). Collective reports to date indicate a need to incorporate typical male symptoms of depression into the current diagnostic criteria for depression, i.e., substance abuse, aggression or violence, stress perception, and emotional suppression, which are suggested by mature questionnaire (112, 119) and hegemonic masculinity (107, 108), and need further validation.

### Encouraging Males to Express Negative Emotions and Seek Help

Empirical evidence indicates that low treatment rates in men cannot be explained by better health, but are instead attributable to a discrepancy between perceptions of need and help-seeking behavior in men. It has been well-documented that males use drugs and alcohol to mask their depression (117, 120, 121). In fact, the common notion that people who seek psychological help because of mental disorders are weak or incapable (122) brings strong stigma to people who seek psychological help, especially for males (108), which prevents them from expressing symptom and seeking help. To fight stigma and prejudice against mental disorders, international campaigns were carried out previously, with a 5.6% increase in people who access mental health services reporting no experienced discrimination, and a fall in average levels of reported discrimination to 28.4% from 41.6% in response to the campaigns (123). These campaigns included an international campaign which is initiated by the World Psychiatric Association (124), “Time to Change” which is launched in 2007 by charities Mind and Rethink Mental Illness (https://www.time-to-change.org.uk/sites/default/files/Stigma%20Shout.pdf), “Heads Together” which is a campaign set up by the Duke and Duchess of Cambridge and Prince Harry in 2016 (https://www.headstogether.org.uk/about-heads-together/), as well as “See Me” which is a similar campaign run in Scotland (https://www.seemescotland.org/). Efforts at the social level deserve continuing to achieve a profound influence and effect.

The social expectation for males (being strong, independent, and exhibiting self-control) holding in different cultures, is contributed to form the traditional masculinity (125), which inhibits the emotional expressiveness and help-seeking behaviors in males (74). Thus, to better diagnose and treat male depression, reversing general expectations shared by the ordinary population toward males might encourage males to seek psychological help, e.g., to allow males to be weak or ill sometimes, and need help occasionally. Moreover, primary healthcare workers, as well as family members should encourage males to open up emotionally and communicate personal feelings of distress (126, 127). Using social media to encourage men with symptoms of depression to seek help should focus on their general trust in doctors, accepting lack of control, and reducing feelings of weakness associated with asking for help (128). Furthermore, as part of recovery from depression, men could reconstruct a valued sense of themselves and their own masculinity, and incorporate values associated with hegemonic masculinity into narratives (re-establishing control, and responsibility to others), which may be useful in reducing depression as well as suicide (129).

### Awareness of Suicidal Behavior in Males With Depression

Studies suggest that undiagnosed and untreated depression in men may be one reason why many more men than women commit suicide (117), since that untreated or inadequately treated depression is the largest risk factor for suicide (63) and 90% of people who die from suicide have a previous psychiatric diagnosis mainly depression (35). Accordingly, increasing the rates of diagnosis and treatment of male depression may be critical to reducing the rate of male suicide. Concerning gender differences in suicidal behaviors, the ingestion of drugs was common for women; and hanging and use of sharp objects for men (130). Moreover, men with depression are less likely to mention suicide before committing suicide (29), rendering male suicide less preventable. Thus, to prevent male suicide more effectively, better recognition of subtle indicators of suicidal thoughts or intentions in males with depression is required.

## Future Studies

The current study is only a theoretical proposal on the “gender differences in self-reporting symptom of depression.” The direct empirical evidence to supporting the above theory is lacking. To test it, more community-based investigations worldwide covering both genders and all age-bands are warranted, since that data from hospitals or private clinics might be biased. The survey tools should be more integrated considering symptoms of male depression. Notably, different degrees of depression (mild, moderate, and severe) and suicide (suicidal ideation, attempted suicide, and committed suicide) need to be clearly classified in the community sample. In addition, study has highlighted the importance of the emotional brain (prefrontal cortex, parietal lobe, central gyrus, and midbrain) in causing depression for females, while the socio cognitive brain (orbitofrontal, posterior, and cingulate cortices; insula) for males (131). These results implied that the medicine or brain stimulation treatment for depression should be adopted differently for female and male patients with depression [also see the findings of “microglia-neuro inflammation-BDNF” interconnection (132)].

## Conclusion

In sum, due to the above-described strong challenges to the female preponderance of depression hypothesis, herein we propose an alternative hypothesis of the gender differences in self-reporting symptom of depression. The main tenets of this alternative hypothesis are that females are more likely to report mild-moderate symptoms of depression, while more severe depression and higher suicide reporting are evident in males. Potential mechanisms behind these observations include covariation between estrogen levels and the incidence peak of female depression, gender differences in coping style, and gender differences in symptom phenotypes. One of the primary aims of developing this hypothesis presented herein is to emphasize that male depression is under-diagnosed and under-treated. To diagnose and treat male depression timely and effectively, it is critical to incorporate male symptoms of depression into the relevant diagnostic criteria, encourage males to express negative emotions, and increase awareness of suicidal behavior in male patients.

## Author Contributions

QD raised the topic and opinion and further revised the manuscript. PS explored the literature and wrote the draft. All authors contributed to the article and approved the submitted version.

## Funding

This work was supported by Key Project of Natural Science Foundation of Chongqing (cstc2020jcyj-zdxmX0009), Medical Innovation Project of Army Medical University (2019ZLX003), and the Key Project and Innovation Project of People's Liberation Army of China (18CXZ005).

## Conflict of Interest

The authors declare that the research was conducted in the absence of any commercial or financial relationships that could be construed as a potential conflict of interest.

## Publisher's Note

All claims expressed in this article are solely those of the authors and do not necessarily represent those of their affiliated organizations, or those of the publisher, the editors and the reviewers. Any product that may be evaluated in this article, or claim that may be made by its manufacturer, is not guaranteed or endorsed by the publisher.

## Footnotes

^1^Both Gotland Male Depression Scale (113, 114) and the Masculine Depression Scale (115) are scales available for evaluating the presence of alternative “male-type” depression symptoms in the clinic. Based on the above two scales, the Male Symptoms Scale (MSS) and the Gender Inclusive Depression Scale (GIDS) (112) have also been published on the given topic. The MSS was developed to assess eight constructs that have been proposed in the literature as externalizing symptoms of depression in men: irritability, anger attacks/aggression, sleep disturbance, alcohol/other drug abuse, risk-taking behavior, hyperactivity, stress, and loss of interest in pleasurable activities (112). The GIDS consisted of 15 symptoms, including the eight constructs contained in the Male Symptoms Scale as well as seven traditional symptoms of depression: sad/depressed mood, loss of vitality, tiredness, ambivalence, anxiety/uneasiness, and “complaintiveness” (feeling pathetic) (112).
